# Pros and cons of tetrastarch solution for critically ill patients

**DOI:** 10.1186/2052-0492-2-23

**Published:** 2014-03-25

**Authors:** Daisuke Toyoda, Shigeo Shinoda, Yoshifumi Kotake

**Affiliations:** Department of Anesthesiology, Toho University Ohashi Medical Center, 2-17-6, Ohashi, Meguro, Tokyo, 153-8515 Japan

**Keywords:** Hydroxyethyl starch, Colloid, Critically ill, Severe sepsis, Acute kidney injury, Coagulopathy

## Abstract

Proper fluid management is crucial for the management of critically ill patients. However, there is a continuing debate about the choice of the fluid, i.e., crystalloid vs. colloid. Colloid solution is theoretically advantageous to the crystalloid because of larger volume effect and less interstitial fluid accumulation, and hydroxyethyl starch (HES) is most frequently used for perioperative setting. Nevertheless, application of HES solution is relatively limited due to its side effects including renal toxicity and coagulopathy. Since prolonged presence of large HES molecule is responsible for these side effects, rapidly degradable HES solution with low degree of substitution (tetrastarch) supposedly has less potential for negative effects. Thus, tetrastarch may be more frequently used in the ICU setting. However, several large-scale randomized trials reported that administration of tetrastarch solution to the patients with severe sepsis has negative effects on mortality and renal function. These results triggered further debate and regulatory responses around the world. This narrative review intended to describe the currently available evidence about the advantages and disadvantages of tetrastarch in the ICU setting.

## Introduction

In the perioperative setting, goal-directed fluid management using hydroxyethyl starch (HES) preparation has been successfully implemented [1, 2]. However, recent randomized controlled studies targeted for critically ill patients demonstrated contradictory results. In this narrative review, advantages and disadvantages of HES preparation, especially most recently developed HES solution with degree of substitution of 0.4 (tetrastarch, HES 130/0.4 or HES 130/0.42), which has low-molecular weight and is rapidly degradable, for fluid resuscitation in ICU or ER setting is discussed.

## Review

The proposed advantages and disadvantages of colloid against crystalloid are summarized in the Table 1[3].

**Table 1:** **Claimed advantages and disadvantages of colloid solution versus crystalloid solution**

Solution	Advantages	Disadvantages
Colloids	Smaller infused volume	Renal dysfunction (dextran > HES > albumin)
	Prolonged increase in plasma volume	Coagulopathy (older HES > tetrastarch > albumin)
	Less peripheral edema	Pulmonary edema (capillary leak syndrome)
	Endothelial protection	Pruritis (HES, dextran > albumin)
		Anaphylaxis (dextran > HES > albumin)
		Greater cost (albumin > other synthetic colloids)
Crystalloid	Lower cost	Short-term increase in intravascular volume
	Greater urinary flow	Short-term hemodynamic improvement
	Interstitial fluid replacement	Interstitial fluid accumulation

Modified from reference [3].

### Characteristics of HES

Starch is a branched polymer of glucose, and it has poor solubility and is rapidly metabolized by α-amylase. To make the starch molecule more soluble and provide clinically relevant persistence in the circulation, some of hydroxyl moiety of starch molecule is substituted with hydroxyethyl residue. The degree of substitution (DS) represents the ratio between hydroxymethylated and unsubstituted portion. C2/C6 ratio represents the position of the carbon atom skeleton of glucose where the substitution predominantly occurs. HES molecule with lower DS and C2/C6 ratio is more susceptible to the effects of amylase and more rapidly eliminated from the circulation than HES molecule with higher DS and C2/C6 ratio [4–6]. The number and the size of the metabolized HES molecule remained in the circulation (*in vivo* molecular weight) play important roles on the volume effects and side effects of the HES solution [7, 8]. Thus, the development has been aimed for HES preparations with low DS, and currently, HES preparation with DS of 0.4 or 0.42 (HES 130/0.4 and HES 130/0.42) is the most advanced solution to date. These solutions are sometimes called tetrastarch according to the number of their DS. Characteristics of several HES preparations as well as other colloids such as albumin and gelatin are summarized in Table 2[9–15]. In this article, the advantages and disadvantages of tetrastarch against other colloids and crystalloids are reviewed.

**Table 2:** **Characteristics of colloid solutions**

Product	Concentration (%)	Oncotic pressure (mmHg)	Initial volume expansion^a^(%)	Persistence in the body (days)	Maximal dose/24 h	Carrier solution	Effect on hemostasis	Comments
Albumin	4	20–29	80	n/a		Na 148 mEq/l	0	
	20	100–120	200 ~ 400			Cl 128 mEq/l		
						Na n/a		
						Cl 19 mEq/l		
Dextran 70	6	56–68	120	28 ~ 42	1.5 g/kg		+++	
Dextran 40	10	168–191	200	6	1.5 g/kg		+++	
Fluid gelatin	4	42	70	2 ~ 7		Na 154 mEq/l	0 ~ +	
			90	7				
						Cl 120 mEq/l		
Urea-linked gelatin	3.5	25–29	70 ~ 80	2 ~ 7		Na 145 mEq/l	0 ~ +	
						Cl 145 mEq/l		
HES 670/0.75	6	25–30	100		20 ml/kg	Lactate Ringer	++(+)	
HES 200/0.5	6	30–37	100	3 ~ 4	33 ml/kg		+	
HES 70/0.5	6		80 ~ 90		20 ml/kg	Either saline or balanced solution	0 ~ +	
HES 200/0.5	10	59–82	145	3 ~ 4	20 ml/kg		+	Used in VICEP study [12]
HES 130/0.4	6	36	100	<1	50 ml/kg	Either saline or similar to acetate Ringer but no Ca	0 ~ +	Used in CHEST study [13] and CRYSTMAS study [14]
HES 130/0.42	6				33 ml/kg	Acetate Ringer		Used in 6S trial [15]

Modified from references [9–11]. HES products are summarized as *in vitro* molecular weight/degree of substitution. ^a^Expressed as plasma volume increase/administered volume (%). The number of plus sign suggests the semi-qualitative comparison between each item.

### Advantages of tetrastarch

#### Smaller infused volume and prolonged increase in plasma volume

Theoretically, colloid solution exerts three to four times larger volume expansion compared to crystalloid solution. This paradigm has been confirmed in healthy volunteers [16], but the volume effect seems much smaller than such theoretical value in the clinical situations. Actually, most of the studies reported that both HES and albumin demonstrated 1.4 to 1.8 times larger volume effects than crystalloid [14, 17–21]. Although some of the authors concluded that the difference was not clinically relevant, we assume that the difference remains clinically relevant since favorable outcome could be achieved with even moderately restrictive fluid regimen in patients with acute respiratory distress syndrome [22, 23].

### Endothelial protection

Several studies demonstrated anti-inflammatory properties and endothelial protection by tetrastarch [24–28]. However, relevant clinical study is not available due to its retraction [29]. Recently, endothelial glycocalyx is recognized to play an important role in the control of vascular permeability [30–33]. *In vitro* model of coronary vasculature, HES 130/0.4 partially attenuated the negative effects of glycocalyx destruction by heparinase [34, 35]. This finding potentially suggests the possible protective effects of tetrastarch molecule in inflammation-related glycocalyx damage.

### Disadvantages of tetrastarch

#### Renal dysfunction

##### Overview

Older HES preparations are known to negatively affect the renal integrity. However, tetrastarch undergoes rapid metabolism and is generally assumed that such side effects are less clinically relevant. However, recent reports suggest even tetrastarch increases the risk of acute kidney injury and renal replacement therapy in ICU patients. In this section, we focused on the interpretation of recent reports about this topic.

### Non-clinical studies

#### In vitro study

*In vitro* study using cultured renal tubular cells demonstrated cytotoxic effect of HES 130/0.4 while crystalloid and albumin demonstrated protective effect [36]. The author estimated that exposure of HES 130/0.4 concentration over 10 mg/ml for more than 4 h may exert deleterious effect on proximal renal tubular cells. Since no metabolism presumably occurs in their experimental setting, this result suggests that prolonged exposure of unmetabolized HES molecule may negatively affect the renal integrity.

### Animal, *in vivo* studies in severe sepsis model

Several animal studies also investigated renal effects of HES 130/0.4 in septic shock model. In rats, HES 130/0.4 negatively affected renal function compared to sham-operated animals [37]. However, interpretation of the data is somewhat difficult since the effects of HES 130/0.4 and crystalloid were not directly compared. In ewes, initial resuscitation of HES and crystalloid resulted similar serum creatinine concentration as well as microscopic finding of renal tubules [38, 39]. These data suggest that resuscitation with HES may not negatively affect renal function in septic shock animals.

### Clinical studies

#### Prospective study in penetrating trauma patients (FIRST study)

In resuscitation of penetrating trauma victims [18], early goal-directed therapy using HES 130/0.4 resulted in milder renal damage than that using saline.

#### Repetitive administration of large dose in traumatic brain injury patients

In traumatic brain injury patients [40], cumulative dose of 19 ± 16 l of HES 130/0.4 (max 66 liter) did not negatively affect creatinine clearance and serum creatinine [40].

### Retrospective study in ICU patients

A retrospective study demonstrated that use of HES 130/0.4 was not a risk factor of acute kidney injury in patients who stayed more than 72 h in the ICU [41].

### Retrospective study in severe sepsis patients

Bayer et al. reported the sequential change of incidence of renal replacement therapy when the principle fluid choice was shifted from HES to gelatin to crystalloid [19, 42] in patients with severe sepsis and postcardiac surgical patients [21]. The authors claimed that the study design was prospective and sequential; we think that the results should be interpreted as a retrospective analysis. They found that the incidence was highest in the period when HES was predominantly used compared to the period when gelatin or crystalloid was used. They concluded that administration of HES compromised renal function and increased the risk of renal replacement therapy. In these studies, the cumulative dose as well as the duration of HES administration was not reported. Thus, there is a fair possibility that HES had been used on multiple days during their ICU stay.

### Prospective, randomized trial in severe sepsis patients (6S trial)

In this randomized, control trial (RCT) [15], 90-day mortality and the incidence of RRT were compared between buffer-based HES 130/0.42 and acetate Ringer solution in severe sepsis patients in ICU. In this trial, HES had been administered 3 days and more in about 50% of the participants and median cumulative dose of HES was reported as 44 ml/kg. Unfortunately, the largest quartiles of cumulative dose are not reported, and the relationship between cumulative dose and outcomes are not analyzed.

### Prospective, randomized trial in ICU patients (CHEST study)

In this RCT [13], 90-day mortality, incidence of acute kidney injury (AKI) and requirement of renal replacement therapy were compared between saline-based HES 130/0.4 and saline. The inclusion criteria were less strict than the 6S trial and ICU patients who had the indication of fluid administration underwent randomization. The percentage of patients with sepsis was about 30% in both groups, and about 15% of the subjects received HES before randomization in both groups. The number of days with HES treatment as well as the cumulative dose was not explicitly described. It is noteworthy that both the HES and saline were liberally administered, and fluid balance was significantly positive especially in the latter part of the study period. There was no difference of 90-day mortality, but the number of patients who underwent renal replacement therapy was marginally but significantly higher in patients assigned to HES group (*p* = 0.04). The subgroup analysis revealed that the HES did not negatively affect the primary outcome in patients with sepsis and AKI before randomization. On the contrary, secondary cardiovascular failure was significantly reduced in patients randomized to HES group.

### Prospective, randomized trial in severe sepsis patients (CRYSTMAS study)

In this RCT [14], hemodynamic effects, incidence of renal injury assessed with risk, injury, failure, loss, end-stage renal disease (RIFLE) criteria as well as several biomarker concentrations were compared between saline-based HES 130/0.4 and saline in severe sepsis patients. Although this study was much smaller than 6S trial and CHEST study, the target of fluid resuscitation and allowable limit of HES were clearly defined as 50 ml/kg on first day and 25 ml/kg afterward. There was no difference of AKI incidence assessed by RIFLE criteria and biomarkers.

### Meta-analysis

This meta-analysis [43] investigated the effects of various HES preparations of HES on renal function. This report concluded that HES was associated with a significant increased risk of mortality and acute kidney injury. This conclusion is derived from the secondary analysis from ten articles including 6S trial, CHEST study, and CRYSTMAS study, but results from studies using different HES preparation were also included [12, 44].

### Prospective, randomized trial in the treatment of hypovolemic shock (CRISTAL trial)

This recent RCT [45] compared colloid and crystalloid on the 28-day mortality, 90-day mortality, renal replacement-free days, ventilator-free days, and vasopressor-free days in patients with hypovolemic shock from various origins. Although this study is not solely focused on HES preparation, the results may be extrapolated to the effects of HES 130/0.4 since it was used in 70% of the colloid group. There was no difference in the 28-day mortality, but most of the secondary outcomes were better with the colloid group.

### Clinical implications of HES-induced renal impairment

According to the *in vitro* study, the prolonged exposure of native HES molecule may be injurious to the renal tissue. Thus, the rapid degradation and elimination of HES molecule may be pivotal to preserve renal integrity. It is yet to be known whether the activity of α-amylase, main metabolic pathway of HES, is intact or impaired in patients with severe sepsis. From this standpoint, the renal damage of HES may be dose-dependent in certain populations and repetitive administration near the upper limit of the maximal dose to the patients with sepsis may not be advisable. Additionally, recent reports highlight the implications of chloride on renal function. This issue is more important for saline-based HES preparation. In volunteers, saline infusion reduced the renal microvascular blood flow compared to balanced solution [46]. Furthermore, chloride-restrictive fluid management reduced renal damage in ICU patients [47], and postoperative hyperchloremia increased the mortality risk in surgical patients [48]. Thus, excessive or liberal administration of chloride may not be also advisable.

### Coagulopathy

#### Overview

Currently, five major pathways have been identified: (1) dilution of coagulation factors, (2) binding and inactivation of factor VIII (fVIII) and von Willebrand's factor (vWF), (3) inhibition of glycoprotein receptor IIb/IIIa (GP IIb/IIIa) on the surface of activated platelet, (4) inhibition of binding between GP IIb/IIIa and vWF or fibrinogen, and (5) acceleration of fibrin degradation [10, 49]. Accordingly, the effects of HES on dilution or binding and inactivation of fVIII or vWF may be evaluated with the plasma concentration of fVIII and vWF [50–53]. Inhibition of GP IIb/IIIa can be quantitated with platelet aggregometry [54], and overall effects may be estimated with viscoelastic analysis of coagulation such as rotational thromboelastometry [55].

Previous data indicate that these effects of HES on coagulation clearly depend on its pharmacokinetic profile, and prolonged presence of large HES molecule supposedly has the large impact on coagulation [56]. Thus, tetrastarch should be least suppressive of coagulation system (Table 2). Furthermore, the presence of calcium in the carrier solution may attenuate the negative impact of HES on coagulation [57]. Unfortunately, most of the currently available data are derived from perioperative setting, and only small numbers of studies from ICU are available.

### Clinical studies

#### Viscoelastic analysis with postcardiac surgery patients

This study compared the effects of 15 ml/kg of HES 130/0.4, HES 200/0.5, and 4% albumin on thromboelastometric tracing in patients after cardiac surgery in the ICU [58]. The author found that clot formation time and maximal clot firmness was decreased immediately after the infusion of both HES preparations. Such changes were partially reversed 2 h after the infusion. On the contrary, albumin did not affect the results of the thromboelastometry. However, they found no difference of the amount of chest tube drainage between the three study groups.

The same authors compared the effects of 28 ml/kg of HES 130/0.4, gelatin, and crystalloid in the similar setting described in the previous paragraph [59]. The author found that clot formation time and maximal clot firmness were decreased in the dose-dependent manner after the infusion of HES 130/0.4 and colloid. Only the changes after HES 130/0.4 infusion were returned to the pre-infusion level. On the contrary, crystalloid slightly but significantly potentiated coagulation. Again, they found no difference of the amount of chest tube drainage between the three study groups.

### Meta-analysis of postcardiac surgical patients

This meta-analysis [60] selected 18 trials to examine the effects of HES on coagulation system in patients undergoing cardiopulmonary bypass. The author found that HES significantly increase the risk of postoperative blood loss and reoperation compared to albumin. The selected studies include the use of different types of HES preparation as well as various clinical contexts such as pump prime, intraoperative fluid administration, and fluid management in the ICU, and therefore, the effects of postoperative use of tetrastarch in the ICU on coagulation is inconclusive. However, the author commented that the analysis did not provide reassurance of a safety profile of tetrastarch due to the fact that sensitivity analysis did not found statistical difference between pentastarch and tetrastarch.

### Repetitive administration of large dose in traumatic brain injury patients

This study is already mentioned in the renal impairment section. Most of the coagulation parameters such as platelet count, fibrinogen concentration, prothrombin time, partial thromboplastin time, and thromboelastographic analysis were comparable between HES 130/0.4 group and HES 200/0.5 supplemented with albumin group. However, plasma concentrations of FVIII and vWF were significantly higher in the HES 130/0.4 group. Such data can be extrapolated as repetitive administration of HES 130/0.4 may not have deleterious effect on coagulation in patients without major predisposing factor of coagulation dysfunction such as postcardiac surgical patients or sepsis [40].

### *Post hoc* analysis of prospective, randomized trial in severe sepsis patients (6S trial)

In this analysis [61], the authors found increased incidence of bleeding in patients who were assigned to HES 130/0.42 group. Multivariate analysis revealed significantly increased risk of any bleeding in patients treated with buffer-based HES 130/0.42 compared to that with acetate Ringer's solution.

### Clinical implications of HES-induced coagulopathy

Most of the previous studies indicate that the persistent presence of large HES molecule in the circulation may be responsible to the HES-induced coagulopathy. Thus, tetrastarch supposedly has more favorable profile on coagulation in patients without underlying coagulation disorders. However, risk–benefit ratio should be carefully evaluated in special populations such as patients after cardiopulmonary bypass and patients with sepsis.

The following interventions may successfully attenuate the effects of tetrastarch on coagulation. First, consider fibrinogen. Although obtained from perioperative setting, abnormalities of maximal clot firmness from rotational thromboelastometry after major bleeding and HES 130/0.4 administration can be successfully reversed by the administration of fibrinogen concentrates [62, 63]. These data intuitively suggest that monitoring and prompt supplementation of fibrinogen is imperative to prevent the consequences of HES-induced coagulation dysfunction. Second, consider supplement of calcium. Adequate ionized calcium is essential for coagulation system. However, saline-based HES 130/0.4, which is currently available in Japan, does not contain calcium in its carrier solution and predisposes the patients to potential hypocalcemia. Although a study with healthy volunteers demonstrated attenuated ADP-induced platelet aggregation and no difference of viscoelastic analysis in saline-based HES 130/0.4 compared to balanced HES 130/0.42 [64], we believe that careful monitoring and timely supplementation of calcium is also essential to attenuate HES-induced coagulopathy.

### Pulmonary edema (capillary leak syndrome)

This is a relatively common concern about colloid administration that extravasated colloid may accentuate interstitial fluid accumulation and worsen pulmonary edema. However, the recent study using extravascular lung water evaluation by transpulmonary thermodilution method failed to substantiate this concern [65].

### Pruritis and anaphylaxis

These issues may also be related to the molecular size of HES, and the incidences of such side effects are relatively low in tetrastarch [5, 66–69]. However, pruritus and skin rashes more frequently occurred in the CHEST study that compared HES 130/0.4 with saline [13].

### Economical and regulatory issues

This issue is dependent on the price of HES, albumin, and crystalloid solution. In Japan, saline-based HES 130/0.4 costs six times higher than typical crystalloid solution, but albumin is approximately ten times more expensive than saline-based HES 130/0.4. Thus, the use of tetrastarch may be economically justifiable in Japan. However, the concern about renal damage caused by HES triggered various responses from each country. For example, European regulatory agency recommends withdrawal of HES preparation and several countries have already implemented such policy. In the US, the authority provided additional warning that made HES contraindicated to septic patients. In Japan, the authority made additional comment into the package insert of HES 130/0.4 basically stating ‘HES 130/0.4 may worsen patients' condition when administered to resuscitate relative hypovolemic state in critically ill patients including severe sepsis. HES 130/0.4 is indicated if therapeutic benefits clearly outweigh such risk’.

## Conclusions

Currently, the advantages of the tetrastarch can be summarized as the following two issues. First, more efficient restoration of circulating blood volume with less interstitial fluid accumulation compared to crystalloid. Second, almost equivalent volume effect can be expected with much less cost compared to albumin. On the contrary, the disadvantage of tetrastarch is possible renal damage when given to critically ill patients for several days. Therefore, maximal advantages can be expected when given to the patients who is hypovolemic not caused by severe sepsis. Furthermore, we believe that it is imperative to define the cumulative dose limit of tetrastarch over several days.

## Abbreviations

DS: degree of substitution
HES: hydroxyethyl starch
RCT: randomized controlled trial
VWF: Von Willebrand's factor.

## References

[1] Gattas DJ, Dan A, Myburgh J, Billot L, Lo S, Finfer S (2012). Fluid resuscitation with 6% hydroxyethyl starch (130/0.4) in acutely ill patients: an updated systematic review and meta-analysis. Anesth Analg.

[2] Van Der Linden P, James M, Mythen M, Weiskopf RB (2013). Safety of modern starches used during surgery. Anesth Analg.

[3] Prough DS, Funston JS, Svensen CH, Wolf SW, Barash PG, Cullen BF, Stoelting RK, Cahalan MK, Stock MC, Ortega R (2013). Fluids, electrolytes, and acid–base physiology. Clinical Anesthesia.

[4] Westphal M, James MF, Kozek-Langenecker S, Stocker R, Guidet B, Van Aken H (2009). Hydroxyethyl starches: different products–different effects. Anesthesiology.

[5] Boldt J (2009). Modern rapidly degradable hydroxyethyl starches: current concepts. Anesth Analg.

[6] Niemi TT, Miyashita R, Yamakage M (2010). Colloid solutions: a clinical update. J Anesth.

[7] Jungheinrich C, Scharpf R, Wargenau M, Bepperling F, Baron JF (2002). The pharmacokinetics and tolerability of an intravenous infusion of the new hydroxyethyl starch 130/0.4 (6%, 500 mL) in mild-to-severe renal impairment. Anesth Analg.

[8] Yamakage M, Bepperling F, Wargenau M, Miyao H (2012). Pharmacokinetics and safety of 6% hydroxyethyl starch 130/0.4 in healthy male volunteers of Japanese ethnicity after single infusion of 500 ml solution. J Anesth.

[9] Ad Hoc Subcomittee of the American Thoracic Society Critical Care Assembly (2004). Evidence-based colloid use in the critically ill: American Thoracic Society Consensus Statement. Am J Respir Crit Care Med.

[10] Van der Linden P, Ickx BE (2006). The effects of colloid solutions on hemostasis. Can J Anaesth.

[11] Myburgh JA, Mythen MG (2013). Resuscitation fluids. N Engl J Med.

[12] Brunkhorst FM, Engel C, Bloos F, Meier-Hellmann A, Ragaller M, Weiler N, Moerer O, Gruendling M, Oppert M, Grond S, Olthoff D, Jaschinsk U, John S, Rossaint R, Welte T, Schaefer M, Kern P, Kuhnt E, Kiehntopf M, Hartog C, Natanson C, Loeffler M, Reinhart K (2008). Intensive insulin therapy and pentastarch resuscitation in severe sepsis. N Engl J Med.

[13] Myburgh JA, Finfer S, Bellomo R, Billot L, Cass A, Gattas D, Glass P, Lipman J, Liu B, McArthur C, McGuinness S, Rajbhandari D, Taylor CB, Webb SA (2012). Hydroxyethyl starch or saline for fluid resuscitation in intensive care. N Engl J Med.

[14] Guidet B, Martinet O, Boulain T, Philippart F, Poussel JF, Maizel J, Forceville X, Feissel M, Hasselmann M, Heininger A, Van Aken H (2012). Assessment of hemodynamic efficacy and safety of 6% hydroxyethylstarch 130/0.4 vs. 0.9% NaCl fluid replacement in patients with severe sepsis: The CRYSTMAS study. Crit Care.

[15] Perner A, Haase N, Guttormsen AB, Tenhunen J, Klemenzson G, Aneman A, Madsen KR, Moller MH, Elkjaer JM, Poulsen LM, Bendtsen A, Winding R, Steensen M, Berezowicz P, Soe-Jensen P, Bestle M, Strand K, Wiis J, White JO, Thornberg KJ, Quist L, Nielsen J, Andersen LH, Holst LB, Thormar K, Kjaeldgaard AL, Fabritius ML, Mondrup F, Pott FC, Moller TP (2012). Hydroxyethyl starch 130/0.42 versus Ringer’s acetate in severe sepsis. N Engl J Med.

[16] Lobo DN, Stanga Z, Aloysius MM, Wicks C, Nunes QM, Ingram KL, Risch L, Allison SP (2010). Effect of volume loading with 1 liter intravenous infusions of 0.9% saline, 4% succinylated gelatine (Gelofusine) and 6% hydroxyethyl starch (Voluven) on blood volume and endocrine responses: a randomized, three-way crossover study in healthy volunteers. Crit Care Med.

[17] Hartog CS, Bauer M, Reinhart K (2011). The efficacy and safety of colloid resuscitation in the critically ill. Anesth Analg.

[18] James MF, Michell WL, Joubert IA, Nicol AJ, Navsaria PH, Gillespie RS (2011). Resuscitation with hydroxyethyl starch improves renal function and lactate clearance in penetrating trauma in a randomized controlled study: the FIRST trial (Fluids in Resuscitation of Severe Trauma). Br J Anaesth.

[19] Bayer O, Reinhart K, Kohl M, Kabisch B, Marshall J, Sakr Y, Bauer M, Hartog C, Schwarzkopf D, Riedemann N (2012). Effects of fluid resuscitation with synthetic colloids or crystalloids alone on shock reversal, fluid balance, and patient outcomes in patients with severe sepsis: A prospective sequential analysis. Crit Care Med.

[20] Bark BP, Persson J, Grande PO (2013). Importance of the infusion rate for the plasma expanding effect of 5% albumin, 6% HES 130/0.4, 4% gelatin, and 0.9% NaCl in the septic rat. Crit Care Med.

[21] Bayer O, Schwarzkopf D, Doenst T, Cook D, Kabisch B, Schelenz C, Bauer M, Riedemann NC, Sakr Y, Kohl M, Reinhart K, Hartog CS (2013). Perioperative fluid therapy with tetrastarch and gelatin in cardiac surgery-a prospective sequential analysis*. Crit Care Med.

[22] Wiedemann HP, Wheeler AP, Bernard GR, Thompson BT, Hayden D, de Boisblanc B, Connors AF, Hite RD, Harabin AL (2006). Comparison of two fluid-management strategies in acute lung injury. N Engl J Med.

[23] Raoof S, Goulet K, Esan A, Hess DR, Sessler CN (2010). Severe hypoxemic respiratory failure: part 2–nonventilatory strategies. Chest.

[24] Lang JD, Figueroa M, Chumley P, Aslan M, Hurt J, Tarpey MM, Alvarez B, Radi R, Freeman BA (2004). Albumin and hydroxyethyl starch modulate oxidative inflammatory injury to vascular endothelium. Anesthesiology.

[25] Feng X, Yan W, Liu X, Duan M, Zhang X, Xu J (2006). Effects of hydroxyethyl starch 130/0.4 on pulmonary capillary leakage and cytokines production and NF-kappaB activation in CLP-induced sepsis in rats. J Surg Res.

[26] Feng X, Yan W, Wang Z, Liu J, Yu M, Zhu S, Xu J (2007). Hydroxyethyl starch, but not modified fluid gelatin, affects inflammatory response in a rat model of polymicrobial sepsis with capillary leakage. Anesth Analg.

[27] Balkamou X, Xanthos T, Stroumpoulis K, Moutzouris DA, Rokas G, Agrogiannis G, Demestiha T, Patsouris E, Papadimitriou L (2010). Hydroxyethyl starch 6% (130/0.4) ameliorates acute lung injury in swine hemorrhagic shock. Anesthesiology.

[28] Heckel K, Winkelmann B, Strunden MS, Basedow A, Schuster A, Schumacher U, Kiefmann R, Reuter DA, Goetz AE (2012). Tetrastarch sustains pulmonary microvascular perfusion and gas exchange during systemic inflammation. Crit Care Med.

[29] Lang K, Suttner S, Boldt J, Kumle B, Nagel D (2003). Volume replacement with HES 130/0.4 may reduce the inflammatory response in patients undergoing major abdominal surgery. Can J Anaesth.

[30] Annecke T, Fischer J, Hartmann H, Tschoep J, Rehm M, Conzen P, Sommerhoff CP, Becker BF (2011). Shedding of the coronary endothelial glycocalyx: effects of hypoxia/reoxygenation vs ischaemia/reperfusion. Br J Anaesth.

[31] Chappell D, Jacob M, Hofmann-Kiefer K, Conzen P, Rehm M (2008). A rational approach to perioperative fluid management. Anesthesiology.

[32] Csete M (2011). New molecular players in the great fluid debate. Anesth Analg.

[33] Collins SR, Blank RS, Deatherage LS, Dull RO (2013). Special article: the endothelial glycocalyx: emerging concepts in pulmonary edema and acute lung injury. Anesth Analg.

[34] Jacob M, Bruegger D, Rehm M, Welsch U, Conzen P, Becker BF (2006). Contrasting effects of colloid and crystalloid resuscitation fluids on cardiac vascular permeability. Anesthesiology.

[35] Jacob M, Bruegger D, Rehm M, Stoeckelhuber M, Welsch U, Conzen P, Becker BF (2007). The endothelial glycocalyx affords compatibility of Starling’s principle and high cardiac interstitial albumin levels. Cardiovasc Res.

[36] Neuhaus W, Schick MA, Bruno RR, Schneiker B, Forster CY, Roewer N, Wunder C (2011). The effects of colloid solutions on renal proximal tubular cells in vitro. Anesth Analg.

[37] Schick MA, Isbary TJ, Schlegel N, Brugger J, Waschke J, Muellenbach R, Roewer N, Wunder C (2010). The impact of crystalloid and colloid infusion on the kidney in rodent sepsis. Intensive Care Med.

[38] Ertmer C, Kohler G, Rehberg S, Morelli A, Lange M, Ellger B, Pinto BB, Rubig E, Erren M, Fischer LG, Van Aken H, Westphal M (2010). Renal effects of saline-based 10% pentastarch versus 6% tetrastarch infusion in ovine endotoxemic shock. Anesthesiology.

[39] Ertmer C, Kampmeier TG, Rehberg S, Morelli A, Kohler G, Lange M, Bollen Pinto B, Hohn C, Hahnenkamp K, Van Aken H, Westphal M (2011). Effects of balanced crystalloid vs. 0.9% saline-based vs. balanced 6% tetrastarch infusion on renal function and tubular integrity in ovine endotoxemic shock. Crit Care Med.

[40] Neff TA, Doelberg M, Jungheinrich C, Sauerland A, Spahn DR, Stocker R (2003). Repetitive large-dose infusion of the novel hydroxyethyl starch 130/0.4 in patients with severe head injury. Anesth Analg.

[41] Boussekey N, Darmon R, Langlois J, Alfandari S, Devos P, Meybeck A, Chiche A, Georges H, Leroy O (2010). Resuscitation with low volume hydroxyethylstarch 130 kDa/0.4 is not associated with acute kidney injury. Crit Care.

[42] Bayer O, Reinhart K, Sakr Y, Kabisch B, Kohl M, Riedemann NC, Bauer M, Settmacher U, Hekmat K, Hartog CS (2011). Renal effects of synthetic colloids and crystalloids in patients with severe sepsis: a prospective sequential comparison. Crit Care Med.

[43] Zarychanski R, Abou-Setta AM, Turgeon AF, Houston BL, McIntyre L, Marshall JC, Fergusson DA (2013). Association of hydroxyethyl starch administration with mortality and acute kidney injury in critically ill patients requiring volume resuscitation: a systematic review and meta-analysis. JAMA.

[44] Schortgen F, Lacherade JC, Bruneel F, Cattaneo I, Hemery F, Lemaire F, Brochard L (2001). Effects of hydroxyethylstarch and gelatin on renal function in severe sepsis: a multicentre randomised study. Lancet.

[45] Annane D, Siami S, Jaber S, Martin C, Elatrous S, Declere AD, Preiser JC, Outin H, Troche G, Charpentier C, Trouillet JL, Kimmoun A, Forceville X, Darmon M, Lesur O, Regnier J, Abroug F, Berger P, Clec'h C, Cousson J, Thibault L, Chevret S (2013). Effects of fluid resuscitation with colloids vs crystalloids on mortality in critically ill patients presenting with hypovolemic shock: the CRISTAL randomized trial. JAMA.

[46] Chowdhury AH, Cox EF, Francis ST, Lobo DN (2012). A randomized, controlled, double-blind crossover study on the effects of 2-L infusions of 0.9% saline and plasma-lyte(R) 148 on renal blood flow velocity and renal cortical tissue perfusion in healthy volunteers. Ann Surg.

[47] Yunos NM, Bellomo R, Hegarty C, Story D, Ho L, Bailey M (2012). Association between a chloride-liberal vs chloride-restrictive intravenous fluid administration strategy and kidney injury in critically ill adults. JAMA.

[48] McCluskey SA, Karkouti K, Wijeysundera D, Minkovich L, Tait G, Beattie WS (2013). Hyperchloremia after noncardiac surgery is independently associated with increased morbidity and mortality: a propensity-matched cohort study. Anesth Analg.

[49] Kozek-Langenecker SA (2005). Effects of hydroxyethyl starch solutions on hemostasis. Anesthesiology.

[50] Langeron O, Doelberg M, Ang ET, Bonnet F, Capdevila X, Coriat P (2001). Voluven, a lower substituted novel hydroxyethyl starch (HES 130/0.4), causes fewer effects on coagulation in major orthopedic surgery than HES 200/0.5. Anesth Analg.

[51] Jungheinrich C, Sauermann W, Bepperling F, Vogt NH (2004). Volume efficacy and reduced influence on measures of coagulation using hydroxyethyl starch 130/0.4 (6%) with an optimised in vivo molecular weight in orthopaedic surgery: a randomised, double-blind study. Drugs R D.

[52] Gandhi SD, Weiskopf RB, Jungheinrich C, Koorn R, Miller D, Shangraw RE, Prough DS, Baus D, Bepperling F, Warltier DC (2007). Volume replacement therapy during major orthopedic surgery using Voluven (hydroxyethyl starch 130/0.4) or hetastarch. Anesthesiology.

[53] Kozek-Langenecker SA, Jungheinrich C, Sauermann W, Van der Linden P (2008). The effects of hydroxyethyl starch 130/0.4 (6%) on blood loss and use of blood products in major surgery: a pooled analysis of randomized clinical trials. Anesth Analg.

[54] Franz A, Braunlich P, Gamsjager T, Felfernig M, Gustorff B, Kozek-Langenecker SA (2001). The effects of hydroxyethyl starches of varying molecular weights on platelet function. Anesth Analg.

[55] Felfernig M, Franz A, Braunlich P, Fohringer C, Kozek-Langenecker SA (2003). The effects of hydroxyethyl starch solutions on thromboelastography in preoperative male patients. Acta Anaesthesiol Scand.

[56] de Jonge E, Levi M (2001). Effects of different plasma substitutes on blood coagulation: a comparative review. Crit Care Med.

[57] Deusch E, Thaler U, Kozek-Langenecker SA (2004). The effects of high molecular weight hydroxyethyl starch solutions on platelets. Anesth Analg.

[58] Schramko AA, Suojaranta-Ylinen RT, Kuitunen AH, Kukkonen SI, Niemi TT (2009). Rapidly degradable hydroxyethyl starch solutions impair blood coagulation after cardiac surgery: a prospective randomized trial. Anesth Analg.

[59] Schramko A, Suojaranta-Ylinen R, Kuitunen A, Raivio P, Kukkonen S, Niemi T (2010). Hydroxyethylstarch and gelatin solutions impair blood coagulation after cardiac surgery: a prospective randomized trial. Br J Anaesth.

[60] Navickis RJ, Haynes GR, Wilkes MM (2012). Effect of hydroxyethyl starch on bleeding after cardiopulmonary bypass: a meta-analysis of randomized trials. J Thorac Cardiovasc Surg.

[61] Haase N, Wetterslev J, Winkel P, Perner A (2013). Bleeding and risk of death with hydroxyethyl starch in severe sepsis: post hoc analyses of a randomized clinical trial. Intensive Care Med.

[62] Fenger-Eriksen C, Jensen TM, Kristensen BS, Jensen KM, Tonnesen E, Ingerslev J, Sorensen B (2009). Fibrinogen substitution improves whole blood clot firmness after dilution with hydroxyethyl starch in bleeding patients undergoing radical cystectomy: a randomized, placebo-controlled clinical trial. J Thromb Haemost.

[63] Fenger-Eriksen C, Tonnesen E, Ingerslev J, Sorensen B (2009). Mechanisms of hydroxyethyl starch-induced dilutional coagulopathy. J Thromb Haemost.

[64] Schaden E, Wetzel L, Kozek-Langenecker S, Thaler U, Scharbert G (2012). Effect of the carrier solution for hydroxyethyl starch on platelet aggregation and clot formation. Br J Anaesth.

[65] van der Heijden M, Verheij J, van Nieuw Amerongen GP, Groeneveld AB (2009). Crystalloid or colloid fluid loading and pulmonary permeability, edema, and injury in septic and nonseptic critically ill patients with hypovolemia. Crit Care Med.

[66] Bork K (2005). Pruritus precipitated by hydroxyethyl starch: a review. Br J Dermatol.

[67] Barron ME, Wilkes MM, Navickis RJ (2004). A systematic review of the comparative safety of colloids. Arch Surg.

[68] Levy JH, Adkinson NF (2008). Anaphylaxis during cardiac surgery: implications for clinicians. Anesth Analg.

[69] Ertmer C, Rehberg S, Van Aken H, Westphal M (2009). Relevance of non-albumin colloids in intensive care medicine. Best Pract Res Clin Anaesthesiol.

